# Functional nanomaterials for enhanced tumor photothermal therapy - the mechanisms and applications

**DOI:** 10.3389/fphar.2025.1604965

**Published:** 2025-07-14

**Authors:** Xiaoling Han, Guohai Feng, Xiaoman Li, Shuyi Mo, Caijing Xu, Jiahui Yan, Li Yu, Rong Zhang, Ying Jin, Xiao Xiao, Li Deng

**Affiliations:** ^1^ Department of Pharmacy, Jilin Medical University, Jilin, China; ^2^ Department of Medical Examination, Jilin Medical University, Jilin, China; ^3^ Department of Cardiovascular Surgery, Gaozhou People’s Hospital, Gaozhou, China

**Keywords:** nanomaterial, polymer nanoparticles, nanophotothermal agent, photothermal therapy, tumor therapy

## Abstract

Photothermal therapy (PTT) offers revolutionary breakthroughs in tumor treatment due to its minimally invasive nature, high selectivity and efficiency. Photothermal therapy is a method of using laser irradiation (near-infrared light) to convert light energy into heat, reaching a relatively high temperature to kill tumor cells. Efficient and stable photothermal conversion materials are the key factors in PTT. There are inorganic and organic nanomaterials used in photothermal therapy. Through chemical modification of them, the functions of targeted drug delivery and combination therapy can be achieved. This work generalizes the features, excellent performance and therapeutic effects of photothermal conversion nanomaterials such as polydopamine (PDA), semiconductor nanoparticles (SNPs), Au nanomaterials, palladium nanosheets (PdNs), and carbon nanomaterials. Their functions and the advantages in photothermal therapy, tumor targeting and inactivation, and the mechanisms of nanophotothermal therapy are summarized. By continuously improving the performance and treatment methods of nanomaterials, more efficient, safe and minimally invasive solutions for tumor treatment are expected.

## 1 Introduction

Many preparations and treatment methods are used to overcome cancer (Liu et al., 2024; Yue et al., 2024). Nanophotothermal agents in photothermal therapy (PTT) convert near-infrared (NIR) light into thermal energy (Liu et al., 2025), directly destroy tumor tissues and efficiently kill them (Ma et al., 2023). Compared with traditional therapies, PTT offers higher localized treatment efficiency (Cui et al., 2022a), targeting tumors more precisely (Shi et al., 2024a). By targeting modification, nanophotothermal agents can actively or passively accumulate in tumor sites (Shi et al., 2024b; Xiong et al., 2024; Ren et al., 2024), enhancing treatment specificity. This specificity allows PTT to eradicate tumors while minimizing damage to normal cells (Komedchikova et al., 2024). Moreover, PTT is a non-invasive or minimally invasive method that avoids surgical incisions or large-scale irradiation (Zhou et al., 2025), reducing discomfort and recovery time of patients (Zheng et al., 2024). So compared to chemotherapy and radiotherapy, PTT has less side effects (Cui et al., 2022b). Additionally, PTT promotes tumor vascular normalization, increasing the accumulation and deep penetration of nanomedicines in tumor tissues (Feng et al., 2024), allowing for synergistic effects when combined with chemotherapy, radiotherapy, or immunotherapy (Xu et al., 2024). Furthermore, PTT induces the local release of tumor-associated antigens (TAAs), which activates the host immune response and reduces the risk of tumor recurrence and metastasis. So combining PTT with immunotherapy create the synergistic immune effect (Fang et al., 2024; Liu et al., 2024). When tumor cells undergo ICD (induced immunogenic cell death) under PTT, they release damage-associated molecular patterns (DAMPs) acting as “danger signals” to recruit dendritic cells (DCs), enhancing tumor antigen presentation, and activating cytotoxic T lymphocytes (CTLs). Furthermore, the localized inflammatory microenvironment generated by PTT can reverse immunosuppressive tumor niches, thereby sensitizing tumors to immune checkpoint inhibitors. This multimodal synergy not only amplifies the direct tumor-killing effects of PTT but also establishes systemic antitumor immunity to suppress distant metastases (Shi et al., 2024). Additionally, by surface modification, nanophotothermal agents can be equipped with imaging function, facilitating tumor diagnosis (Zhang et al., 2024). In summary, nanophotothermal tumor therapy exhibits remarkable advantages such as efficiency, specificity, minimal invasiveness, low adverse reactions, multifunctionality and synergy (Lu et al., 2023; Lian et al., 2021; Wang et al., 2024). Nonetheless, clinical application still faces critical issues to be addressed. Approved experimental PTT nanosystem gold nanoshell (AuroShell) NCT02648035 has demonstrated the precision of local thermal ablation in clinical trials of head and neck cancer, but the recurrence rate after a single treatment (−30%) suggests the need for combination chemotherapy or immunotherapy to improve long-term efficacy (Rastinehad et al., 2019). Carbon based materials, such as graphene oxide, exhibit low systemic toxicity in the treatment of melanoma (NCT04323020), but are limited by the depth of light penetration (<2 cm), resulting in insufficient efficacy for deep tumors (Zhao et al., 2023). The major clinical application bottlenecks include (a) biosafety: some metal based nanomaterials (such as CuS, Fe_3_O_4_) have not yet undergone long-term toxicological evaluation by FDA/EMA due to their potential toxicity caused by long-term retention; (b) lack of standardized parameters: the clinical protocol for laser power density (0.3–2 W/cm^2^) and irradiation time (1–10 min) lacks a unified standard, resulting in significant fluctuations in therapeutic efficacy. It is inferred that precise delivery, controllable release and multimodal combination therapy are the keys for technological optimization and future direction of nano PTT.

## 2 Nanomaterials for photothermal therapy

The nanomaterials in PTT are mainly divided into two categories: inorganic and organic nanomaterials. Inorganic nanomaterials were studied earlier, mainly including noble metal nanoparticles, metal chalcogenide nanomaterials, carbon based nanomaterials, magnetic nanoparticles and quantum dots. Precious metal nanoparticles such as gold, silver, platinum and palladium, are commonly used in tumor PTT. Other kind of inorganic nanomaterials such as tungsten nitride (WN) nanoparticles and carbon nanospheres have also been used. Compared to inorganic nanomaterials, the research on organic nanomaterials is relatively limited. Organic nanomaterials typically have good biocompatibility and degradability, but may not be as efficient and stable in photothermal conversion as inorganic nanomaterials. The followings provide detailed introductions to photothermal conversion nanomaterials of polydopamine (PDA), semiconductor nanoparticles (SPNPs), AuNPs, palladium nanosheets and carbon nanomaterials. The performance comparisons of different nanomaterials are listed in Tables 1, 2 shows the applications of the nanomaterials discussed in this review.

**TABLE 1:** Key performance metrics of nanomaterials in photothermal cancer therapy.

Material	Morphology	Absorption peak (nm)	Photothermal efficiency (%)	Advantages	Challenges	Targeted applications	References
Polydopamine (PDA)	Spherical nanoparticles	700–850 (NIR-I)	35–50	Biodegradable High drug-loading capacity	Low photostability Weak NIR-II response	Theranostic platforms	Lu et al. (2021), Balavigneswa et al. (2024), Feng et al. (2024b), Jie et al. (2023), Xu et al. (2022), Wang et al. (2022a), Xu et al. (2023)
Semiconductor NPs (SNPs)	Quantum dots Nanorods	800–1,300 (NIR-I/II)	40–75	Tunable bandgap ROS generation capability	Potential heavy metal toxicity	Image-guided therapy	Terna et al. (2021), Peng et al. (2019), Lyu et al. (2018)
Gold NPs (AuNPs)	Nanoshells Nanorods	700–1,100 (NIR-I/II)	25–60	Surface plasmon resonance Easy functionalization	High cost Limited penetration depth	Surface-enhanced PTT	Ferreira-Gonçalves et al. (2023), Yang et al. (2023a), Kim et al. (2022), Camacho et al. (2022), Yang et al. (2024), Zhang et al. (2020)
Palladium Nanosheets	2D ultrathin sheets	1,000–1,350 (NIR-II)	60–85	Deep tissue penetration High photostability	Complex synthesis Long-term biosafety concerns	NIR-II-driven therapy	He et al. (2022), Yao et al. (2022), Li et al. (2022a)
Carbon Nanomaterials	Graphene, CNTs Nanodiamonds	600–1,200 (Broadband)	30–70	Multifunctional (e.g. drug delivery imaging)	Poor dispersibility Inhomogeneous heating	Hyperthermia Drug delivery	Sajja et al. (2021), Afroze et al. (2021), Farbod et al. (2024), Mohanta et al. (2019)
Silver NPs (AgNPs)	Spherical Triangular plates Nanowires	400–800 (Visible-NIR-I)	50–70	High plasmonic activity Antibacterial	Cytotoxicity Oxidation instability	Localized tumor ablation	Zhao et al. (2022), Moonshi et al. (2023), Liu et al. (2022a)
Tungsten Nitride (WN)	Spherical Porous 2D ultrathin sheets	900–1,100 (NIR-II)	40–60	High photostability Synergistic PTT/PDT	Large-scale synthesis protocols Limited *in vivo* studies	Deep-tissue penetration	Fernandes et al. (2020), Xu et al. (2019)
Platinum NPs (PtNPs)	Spherical Cubic/Octahedral Porous	700–1,000 (NIR-I)	30–50	Catalytic activity ROS generation	High cost Low biocompatibility	Combinational therapy (PTT/chemo)	Runzan et al. (2023), Liu et al. (2024c), Ying et al. (2025)

**TABLE 2:** The structure characteristics, treatment mechanism and clinical applications of PDA, SNPs, Au nanomaterials, PdNs and carbon nanomaterials.

Material	Structure Characteristics	Treatment mechanism	Application cases	Clinical progress	References
Polydopamine (PDA)	High biocompatibility, easy surface functionalization, capable of loading drugs/genes	Photothermal conversion (moderate efficiency), synergistic chemotherapy/immunotherapy	Treatment of melanoma; Drug delivery synergistic PTT (such as doxorubicin)	Theranostic platforms	Xu et al. (2022), Wang et al. (2022a), Xu et al. (2023)
Semiconductor NPs	Adjustable bandgap and strong photoresponsiveness	Photothermal/Photodynamic synergy (high photothermal efficiency)	Breast cancer (NIR-II challenge); Combination radiotherapy/chemotherapy for deep tumor treatment	Image-guided therapy	Terna et al. (2021), Peng et al. (2019), Lyu et al. (2018) Li et al. (2023a), He et al. (2020), Pan et al. (2022), Yao et al. (2023), Alamdari et al. (2022), Yuan et al. (2021), Chen et al. (2020)
Gold NPs (AuNPs)	Strong surface plasmon resonance effect	Photothermal ablation (high efficiency, >90%)	Local ablation of head and neck cancer; Gold nano shell for image-guided PTT	Surface-enhanced PTT	Ferreira-Gonçalves et al. (2023), Yang et al. (2023a), Kim et al. (2022), Camacho et al. (2022), Yang et al. (2024) Zeng et al. (2025), Kim et al. (2021a), Fan et al. (2023), Guerrero-Florez et al. (2020)
Palladium Nanosheets	Ultra thin two-dimensional structure, high specific surface area, surface plasmon resonance	Efficient photothermal conversion (>80%), catalytic activity	Drug resistant tumor treatment; Combined catalytic therapy (such as producing reactive oxygen species)	NIR-II-driven therapy	He et al. (2022), Yao et al. (2022), Li et al. (2022a) Sathiyaseelan et al. (2021), Singh et al. (2023)
Carbon Nanomaterials	High thermal conductivity, chemically inert	Photothermal ablation (efficient, >60%), assisted by photoacoustic imaging	Local treatment of skin cancer;Composite iron oxide nanoparticles for combined magnetic thermal PTT therapy	Hyperthermia Drug delivery	Sajja et al. (2021), Afroze et al. (2021), Farbod et al. (2024), Mohanta et al. (2019) Amaral et al. (2021), Shima et al. (2023)

### 2.1 Polydopamine (PDA)

**FIGURE 1:**
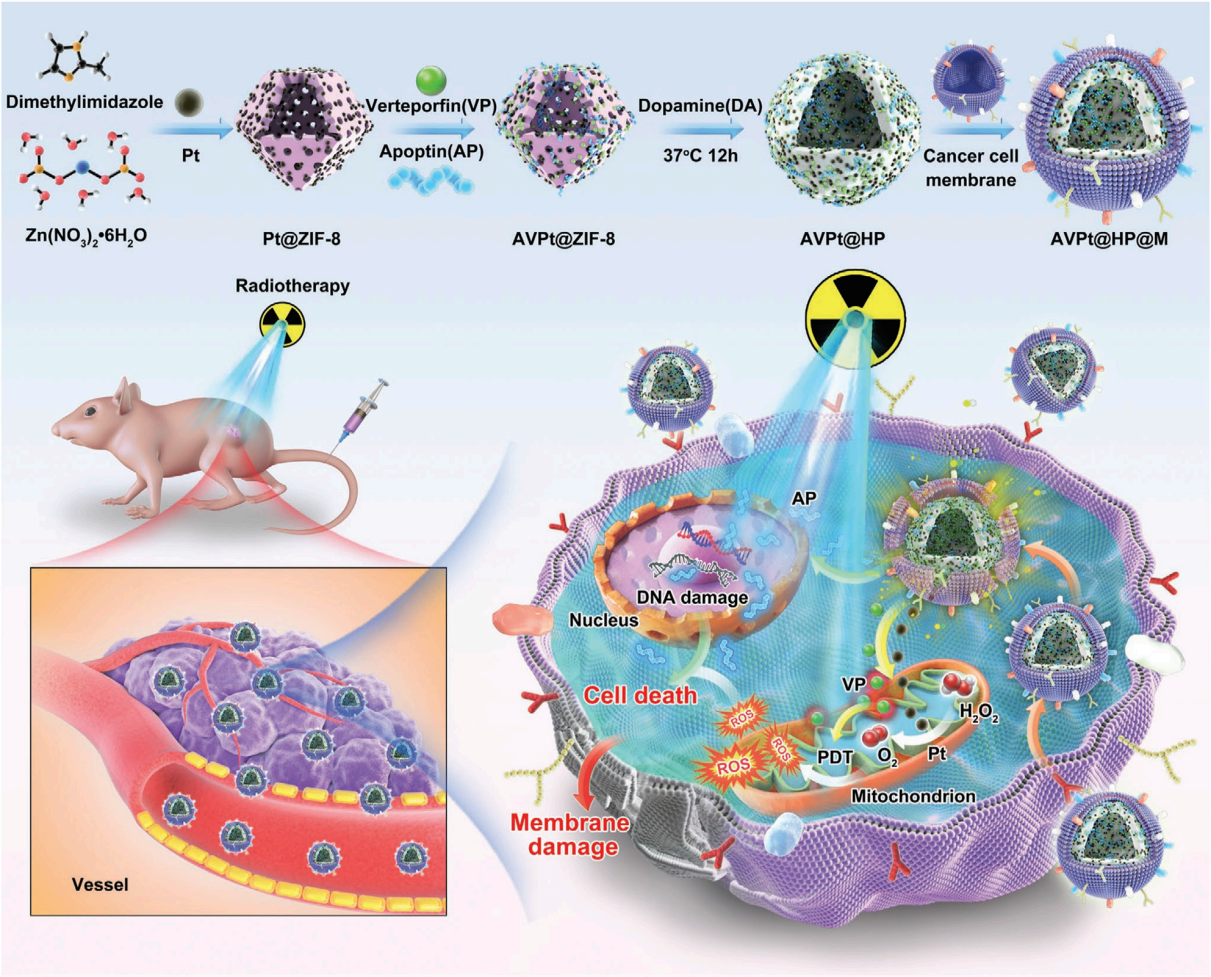
Schematic illustration of biomimetic nanoplatform AVPt@HP@M for colon cancer radiotherapy sensitization through hypoxia relief (Pt-doped ZIF-8), apoptin-enhanced apoptosis (AP), and VP-mediated X-PDT, with cancer cell membrane camouflage for targeted delivery (reprinted with permission from Gong et al., 2022. Copyright 2022 Wiley - VCH GmbH).

PDA possess a unique core-shell structure, and the surfaces are enriched with active functional groups such as phenolic hydroxyl and amino groups (Lu et al., 2021). These groups confer excellent modification and multifunctionality to PDA (Balavigneswa et al., 2024). PDA exhibits strong adhesion, similar to the foot threads of natural mussels (Feng et al., 2024), allowing it to firmly adhere to almost any material surface, forming a conformal layer (Jie et al., 2023). *In vivo*, PDA demonstrates good biocompatibility and limited biological toxicity, which makes it suitable for biomedical applications (Xu et al., 2022). The photothermal effect of PDA originates from the broadband optical absorption (300–900 nm) of its polyphenol-quinone conjugated structure. Under NIR excitation, electrons within the π-π stacking layers convert photon energy into lattice vibrations (phonons) via non-radiative relaxation, releasing thermal energy (Wang et al., 2022). The hydrogen-bonding network further enhances photothermal conversion efficiency through intermolecular vibrational coupling. Surface amino modification (e.g. RGD peptides) enables targeted binding to integrin receptors (e.g., αvβ3) overexpressed on tumor cells, facilitating clathrin-mediated endocytosis. Following internalization, PDA accumulates in lysosomes (pH 4.5–5.0), where its alkaline groups induce lysosomal membrane permeabilization (LMP), releasing heat shock protein 70 (HSP70) and activating apoptosis pathways. PDA degradation products (e.g., dopamine monomers) suppress M2 polarization of tumor-associated macrophages (TAMs), synergistically enhancing antitumor immune responses (Xu et al., 2023). The schematic diagram of the process of using DPA based biomimetic nanomaterials for PTT and killing colon cancer is shown in Figure 1 (Gong et al., 2022), which has a pH-responsive property and can be degraded in a weakly acidic tumor microenvironment (TME), leading to loaded drug release, and the released Apoptin (AP) can work as a radiosensitizer to improve the RT and destroy tumor cells by promoting apoptosis directly. Researchers have constructed many PDA drug delivery systems that simultaneously encapsulate photosensitizers and antitumor drugs within, enabling targeted delivery (Zhang et al., 2022). Zeng addressed the issue of tumor hypoxia by using MnO_2_ nanoparticles loaded with chlorin E6 (Ce6) and coated with folic acid-functionalized PDA layer (MCPFNP) (Zeng et al., 2020). Due to the active targeting mediated by folic acid and the passive transport of enhanced permeability and retention (EPR) effect, MCPFNP significantly accumulated in the tumor sites of mice. In the acidic tumor microenvironment, PDA disassembled and released the photosensitizer Ce6, completing the precise tumor targeting. Simultaneously, under 808 nm laser irradiation, the system generated high temperatures and burned tumor cells, which reduced MCP-7 cell viability to 11.74%. MCPFNP also exhibited excellent biodegradability and low long-term toxicity. Zhang and Wang synthesized a PDA nanomaterial (PTTPB) with PDA and tributyl tetradecylphosphonium bromide (TTPB) (Li et al., 2022), which similarly got precise tumor treatment. PTTPB can recognize sialic acid (SA), which is underexpressed in normal cells and overexpressed on the surface of tumor cells such as B16 F10, thus it targets tumor cells. Upon irradiation, PTTPB elevated the temperature through photothermal effect and ablate the tumor tissue. Additionally, the donor-acceptor (D-A) electronic structure of PTTPB generated singlet oxygen and other reactive oxygen species (ROS) under light, further killing tumor cells, achieving combined photothermal-photodynamic therapy, and significantly improving therapeutic efficiency. Biocompatibility tests showed that PTTPB exhibited low cytotoxicity and good biosafety. Activating the interferon gene stimulating factor (STING) pathway is a highly promising approach for tumor treatment, but its clinical application is limited by the inability to administer systemically and the immunosuppressive tumor microenvironment (TME). Zeng constructed a PDA multifunctional platform loaded with the STING agonist methylated adenylate-2 (MAS-2) and chelated Mn^2+^ with mesoporous PDA (Zeng et al., 2023). This system accumulated in the tumor region through the EPR effect and performed PTT on tumor under NIR irradiation, inducing apoptosis of tumor. During the process, tumor cells released TAAs and pro-inflammatory factors, alleviating the immunosuppressive TME. The platform released MAS-2 and Mn^2+^, activating the STING pathway, ultimately triggered a strong immune response and showed high anticancer effects. In summary, the innovative design of PDA materials in PTT can significantly enhance their functionality. The latest technologies include: Ⅰ. Multifunctional composite carriers, such as gold nanorods or carbon dots wrapped in PDA, to enhance photothermal conversion efficiency (up to 60% or more) and integrate drug delivery/imaging functions; Ⅱ. Surface engineering involves modifying targeted ligands (such as folate) with amino or carboxyl groups to enhance tumor selectivity; Ⅲ. Responsive release system utilizes the pH/ROS sensitivity of PDA to achieve controlled drug release. The challenges lie in long-term biosafety, large-scale synthesis stability, and limitations on deep tissue penetration. Future development will focus on synergistic therapies (such as PTT/chemotherapy/immunotherapy), development of biodegradable PDA derivatives, and AI assisted material design to promote clinical translation, optimize performance balance, and address metabolic mechanism issues.

### 2.2 Semiconductor nanoparticles (SNPs)

**FIGURE 2:**
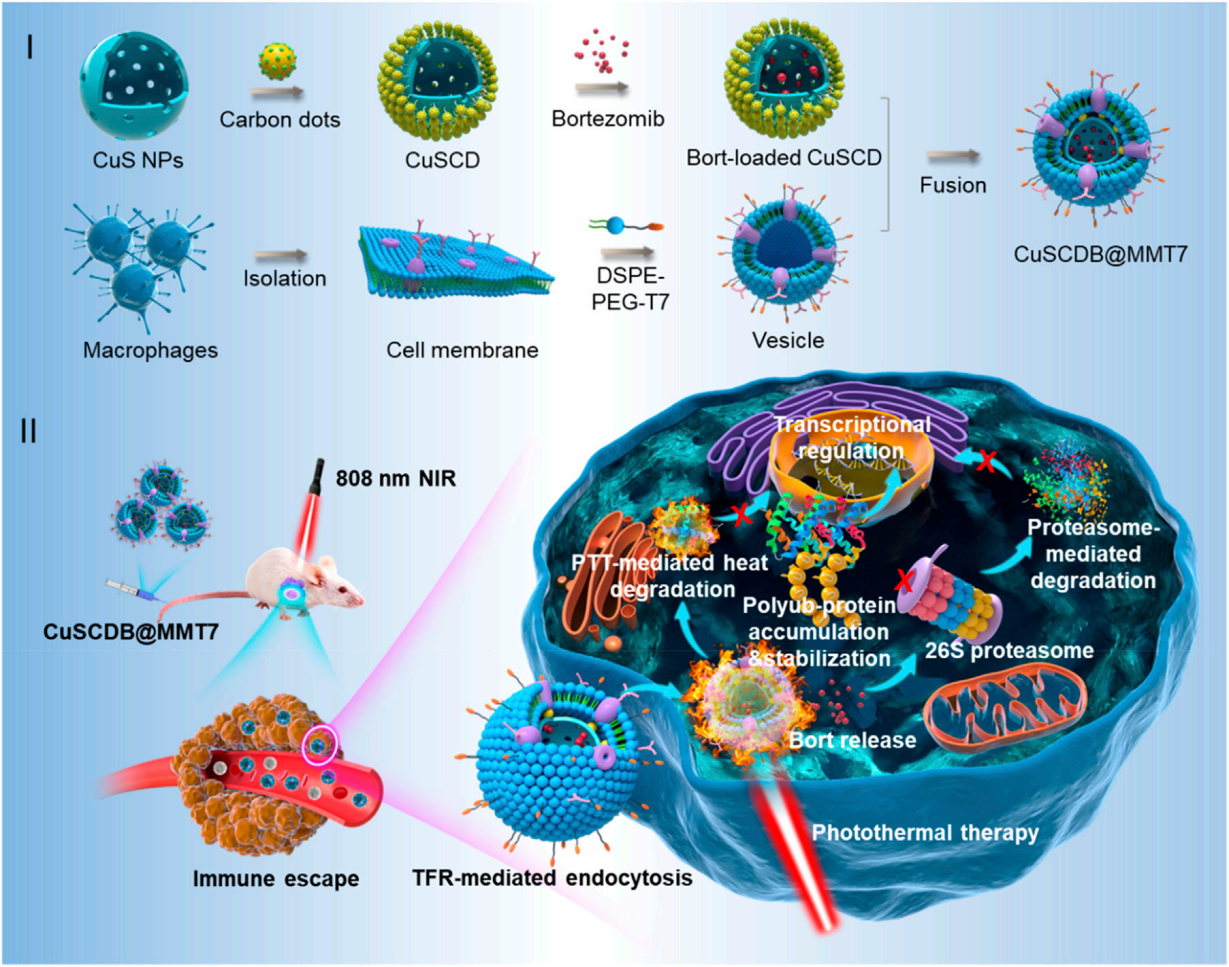
Schematic illustration of the generation of proteasome inhibitor-encapsulated CuS/carbondots nanocomposites for enhanced photothermal therapy viaheat-stabilization of varioussubstrates in the ubiquitin-dependent proteasomal degradation pathway (reprinted with permission from Yu et al., 2020. Copyright 2020 American Chemical Society).

The core structure of SNPs determines their fundamental properties (Terna et al., 2021), while the shell serves to protect the core, enhance optical properties, and improve stability (Peng et al., 2019). Unlike precious metal materials, the ion release properties of SNPs endow them with the potential for photothermal chemodynamic combined therapy (Zhang et al., 2024). The photothermal effect of SNPs comes from bandgap modulation and free carrier oscillation. Narrow bandgap design allows NIR light to excite electrons from the valence band to the conduction band, and then convert energy into heat through “electron phonon scattering” (Li et al., 2023). Sulfur vacancies or oxygen doping (such as MoS_2-x_O_x_) can form intermediate energy levels, enhancing non radiative recombination pathways (carrier lifetime<1ns). SNPs enter cells through caveolae mediated endocytosis, and particles smaller than 50 nm in size can penetrate the nuclear membrane. The local high temperature (ΔT>10°C) generated by photothermal stimulation triggers the release of metal ions from SNPs through Fenton reaction to generate hydroxyl radicals (·OH), leading to mitochondrial DNA damage (Li et al., 2021). Figure 2 shows a schematic diagram of hollow structured CuS NPs composite with carbon dots (CuSCDs) enhancing PTT through ubiquitin dependent proteasome degradation pathway (Yu et al., 2020). SNPs remain stable in biological systems, resist to aggregation or degradation, and exhibit low biotoxicity, ensuring effective PTT with minimal side effects (Zhao et al., 2022). The surface of SNPs have unsaturated coordination, resulting in numerous surface defects and active sites which are modifiable to achieve multifunctionality, such as targeted delivery and biocompatibility (Peng et al., 2019). For instance, Yan Lyu developed a water-soluble SNP (SPNV) through vinylene bonds through simple chemical reactions and physical cross-linking processes (Lyu et al., 2018). SPNV owns the mass absorption coefficient (1.3-fold) and PCE (2.4-fold). The study revealed that the vinylene bonds enhance the biodegradability and optical activity of SNPs, while also improving their imaging and therapeutic capabilities. Thus, appropriate chemical modifications can further expand the biomedical applications of SNPs (Kshatriya et al., 2024), including drug delivery and PTT (Alghamri et al., 2022). In tumor vascular disruption therapy, the off-target effects and repeated dose toxicity of vascular disrupting agents (VDAs) limit overall therapeutic efficacy. To solve this problem, Li constructed biomimetic SNPs containing surface-modified platelet membranes (Li et al., 2023). The SNPs are capable of precise tumor vascular disruption via two-stage light manipulation. During the first irradiation, the nanoparticles generated mild heat to induce tumor vascular bleeding, activating the coagulation cascade and recruiting more nanoparticles to the damaged vessels. During the second irradiation, enhanced targeting of tumor vasculature by the photothermal agents led to intense hyperthermia effectively, destroying the tumor vasculature and completely eradicating the tumor, while also inhibited metastasis. He reported iron-chelated SPFeN composed of ferroptosis inducers (Fe^3+^) and amphiphilic semiconductor copolymers (SPC) (He et al., 2020). Upon NIR irradiation, localized heating occurred and these particles accelerated the Fenton reaction to generate free radicals, assisting tumor suppression and achieved combined PTT-photodynamic therapy. In the acidic tumor microenvironment, SPFeN generated hydroxyl radicals, leading to ferroptosis. Compared to previous studies, SPFeN-mediated ferroptosis PTT can minimize iron dosage and effectively inhibit tumor growth *in vivo*. Additionally, Pan synthesized similar nanoparticles, gadolinium-containing SPN-Gd which exhibited significant inhibitory effects on oral squamous cell carcinoma (OSCC) (Pan et al., 2022). *In vivo*, SPN-Gd as an MRI contrast agent and optical imaging agent, showed a prolonged retention time and significantly inhibited OSCC tumors in mouse models through PTT. The latest technologies for enhancing the therapeutic effect of SNPs include: Ⅰ. Band engineering, which enhances NIR absorption by adjusting the bandgap (such as doping or heterostructure design); Ⅱ. Multi modal collaboration, combined with PDT or chemodynamic therapy (CDT), such as MoS_2_ loaded Fe^2+^ to achieve PTT/CDT (Zhang et al., 2020); Ⅲ. Surface functionalization, such as polyethylene glycol (PEG) modification to enhance biocompatibility, or coupling antibodies to enhance targeting. The challenges of SNPs lie in long-term toxicity, photothermal stability and deep tissue penetration efficiency. Developing new narrow bandgap semiconductors (such as organic-inorganic hybrid perovskites), intelligent responsive nanosystems (such as photo/thermal dual controlled release drugs) and clinical translational research can promote precise PTT tumor treatment.

### 2.3 Au nanoparticles and nanorods

Au nanomaterials exhibit strong light absorption ability, and their photothermal effect originates from localized surface plasmon resonance (LSPR). When the frequency of incident light matches the oscillation frequency of free electrons on the material surface, a strong electromagnetic field is generated, and energy is converted into hot electrons through Landau damping, followed by the release of thermal energy through electron phonon scattering. Due to the influence of the specific surface area, size and shape of Au nanomaterials on their PTT performance, different shapes of Au nanomaterials have been developed, such as Au nanoparticles (AuNPs) and Au nanorods (AuNRs) (Zhao et al., 2022). The longitudinal LSPR of AuNRs can be adjusted to the NIR-II region (1,000–1,350 nm), and the larger the aspect ratio (AR), the deeper the penetration depth. The surface charge of Au nanomaterials (such as CTAB modified positive charges) can disrupt the lipid bilayer structure of tumor cell membranes, leading to increased membrane permeability and calcium ion influx. Accumulation of gold nanomaterials in lysosomes inhibit the mTOR pathway, promote autophagosome formation, and antagonize photothermal induced apoptosis. It is necessary to combine autophagy inhibitors (such as chloroquine) to enhance therapeutic efficacy (Wei et al., 2022).

#### 2.3.1 Au nanoparticles (AuNPs)

**FIGURE 3:**
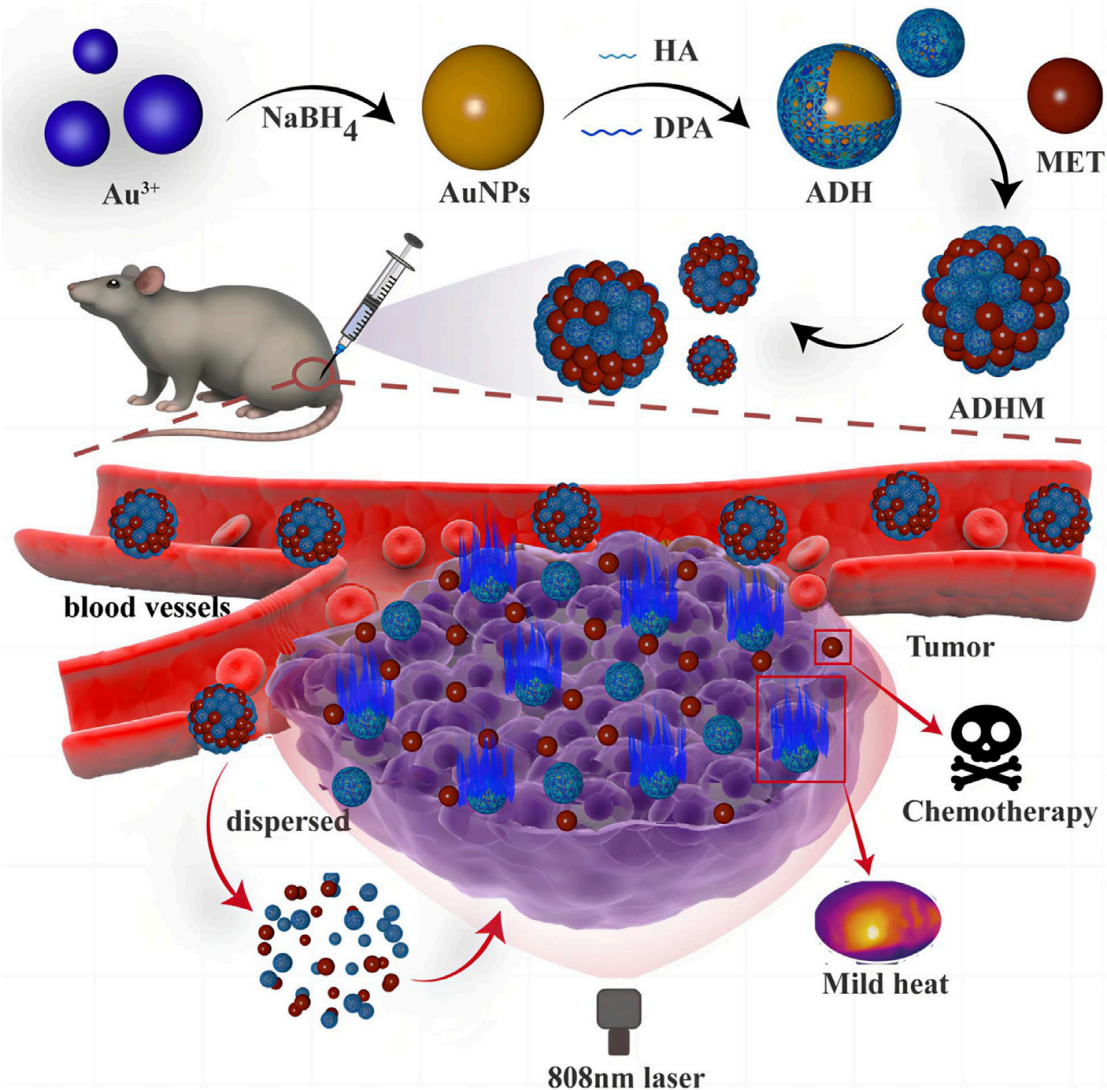
Scheme of mild chemo-photothermal synergistic therapy based on gold nanopaticles coupled with metformin (reprinted with permission from Yang et al., 2023. Copyright 2023 American Chemical Society).

AuNPs demonstrate good PCE (Ferreira-Gonçalves et al., 2023), localized surface plasmon resonance (LSPR) absorption (Yang et al., 2023), high transport efficiency and supramolecular recognition ability (Kim et al., 2022), enabling them to precisely adsorb proteins and target macromolecules within cells or on cell surfaces (Camacho et al., 2022). However, for the reason that working temperature during photothermal therapy is often high (>50°C), surrounding tissues near the tumor are frequently burned, limiting the further application of chemotherapy-photothermal combination therapies (Yang et al., 2024). For that, Yu designed a mild AuNPs photothermal agent (ADHM), composed of dopamine (DA) and hyaluronic acid (HA)-coated AuNPs, assembled with metformin (MET) via electrostatic interactions (Figure 3) (Yang et al., 2023). As shown in Figure 3, ADHM selectively accumulates in tumors via HA-mediated active targeting and the EPR effect, followed by pH-responsive dispersion. The 808 nm NIR-induced mild hyperthermia then synergizes with MET therapy for enhanced antitumor efficacy. This system achieved 94.6% inhibition rate for chemotherapy-photothermal combination therapy of 4T1 tumors in mice at a mild temperature (43°C) while effectively preventing tumor metastasis. The chemical interactions within ADHM exhibit a high degree of synergy, bringing the system excellent targeting ability and biocompatibility. ADHM provides a potential candidate for mild chemotherapy-photothermal combination therapy of tumors. To extend drug accumulation and retention within tumor cells, and enhance the therapeutic efficacy of tumor photothermal therapy, the modifications of surface ligand types, charge, chemical polarity, chemical reactivity, and hydrophobicity of AuNPs photothermal agents (PTAs) are usually used. Wang prepared a mixed-charge zwitterionic surface Au nanoparticle (Au-MUA-TMA) with chemical modification and altered the shape and size of the AuNP to shift the light absorption to the near-infrared (NIR) region (Kong et al., 2023). Under 808 nm laser irradiation, Au-MUA5-TMA5 targeted and accumulated at the U87MG tumor site in mice, causing a significant increase in local temperature and leading to complete tumor disappearance within 14 days of treatment.

#### 2.3.2 Au nanorods (AuNRs)

**FIGURE 4:**
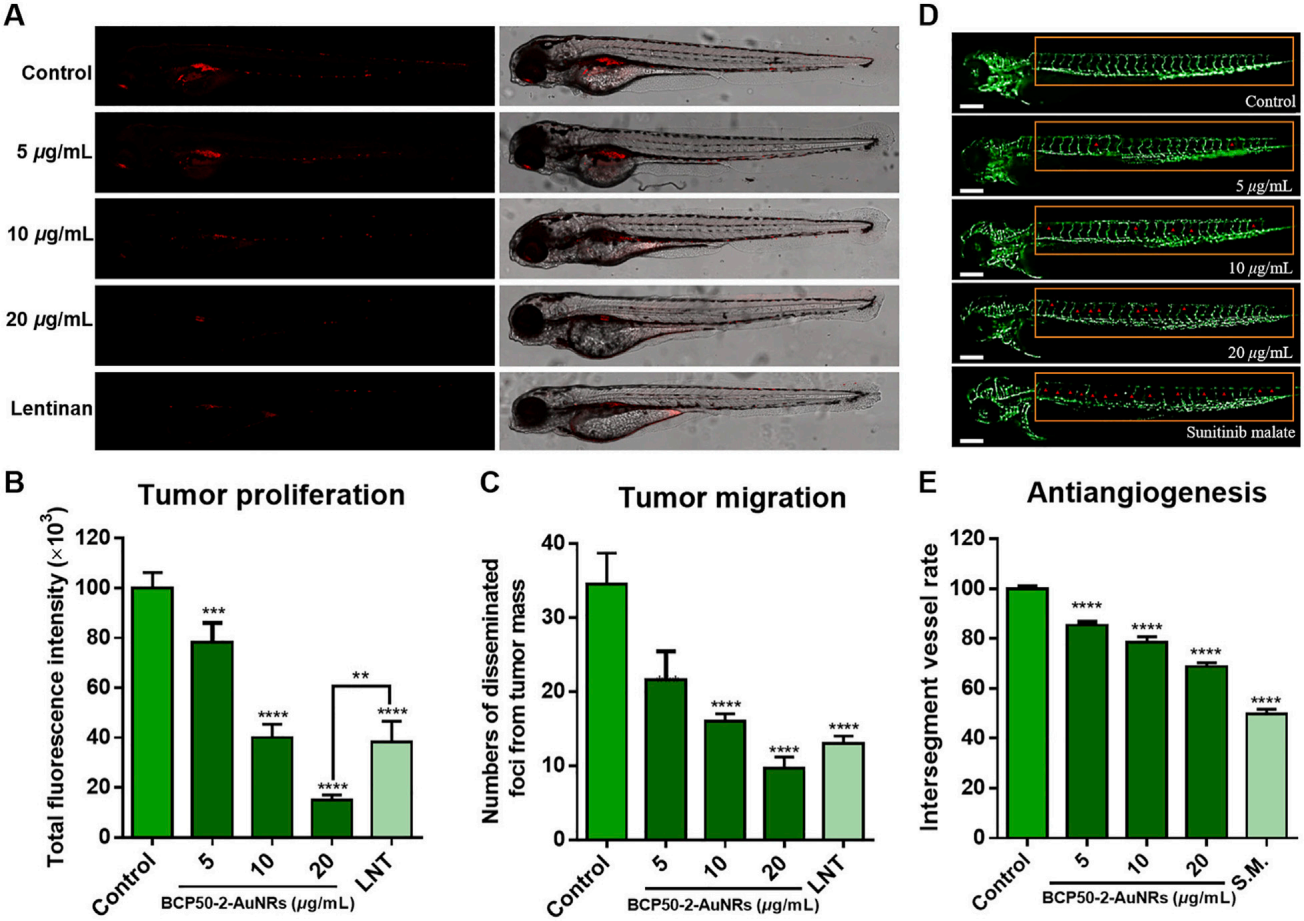
*In vivo* antitumor activity of BCP50-2-AuNRs. **(A)** Confocal images of HepG2-xenografted zebrafish treated with BCP50-2-AuNRs (vs. Lentinan, 400 μg/mL). **(B,C)** Quantification of tumor fluorescence intensity and foci (n = 20 in each group). **(D)** Intersegmental vessels development in transgenic zebrafish (vs. sunitinib malate, 1 μg/mL). **(E)** Angiogenesis inhibition rate (n = 20 in each group) (reprinted with permission from Zhao et al., 2024a. Copyright 2024 Elsevier B.V.).

In contrast to the spherical structure of AuNPs, AuNRs are elongated and rod-like, which makes AuNRs more prone to absorb and scatter light in the long-wavelength region (Roach et al., 2021). Similar to AuNPs, AuNRs also exhibit excellent optical absorption and scattering cross-sections, PCE, LSPR and their surface is easy to be functionalized (Dong et al., 2024). Compared to zero- and one-dimensional nanomaterials, two-dimensional nanomaterials offer significant advantages in PTT-based tumor therapies, such as ultra-thin structures, high specific surface areas and unique optical properties (Wu et al., 2022). For example, Kong combined 2D peptide nanosheets (PNS) with AuNRs to form PNS-AuNRs (Cheong et al., 2021). Upon irradiation, MTT assays on human breast cancer cells and MCF-7 cells showed more than 75% inhibition rates. PNS-AuNRs were also tested *in vivo* for PTT on mice bearing MCF-7 tumors. Within 10 min of irradiation, the tumor cells underwent apoptosis. After 14 days, tumor disappeared completely. Meanwhile, the mice exhibited good health, which demonstrates the low toxicity and general anticancer effects of AuNRs. AuNRs show two characteristic light absorption peaks due to the SPR effects on transverse and longitudinal surfaces. Cheong adjusted the aspect ratio of AuNRs to shift the light absorption peak to NIR region and achieved effective PTT (Figures 4A–C) (Zhao et al., 2024a). The anti-angiogenic effects of BCP50-2-AuNRs were evaluated in Tg (fli1:EGFP) zebrafish. BCP50-2-AuNRs suppressed angiogenesis in a dose-dependent manner (14.6% at 5 μg/mL, 21.5% at 10 μg/mL, and 31.2% at 20 μg/mL) (Figures 4D,E). Some nanomaterials are unstable in physiological environments and tend to aggregate and form precipitates. To improve the stability and antitumor activity, Zhao extracted a homogeneous polysaccharide (BCP50-2) from Belamcanda (a plant) and conjugated it with AuNRs to produce BCP50-2-AuNRs (Kong et al., 2024). BCP50-2-AuNRs exhibited good stability and PCE. Under NIR irradiation, the local temperature of HepG2 tumors increased significantly with BCP50-2-AuNRs and the tumor cell growth were inhibited efficiently. Moreover, the biological models showed good growth without much side effect.

In summary, innovative strategies to enhance the efficacy of AuNPs and AuNRs include the following: Ⅰ. Morphology optimization: adjusting the aspect ratio of Au NRs (such as 50–70 nm length) to match the near-infrared (NIR) window and improve photothermal conversion efficiency (>90%); Ⅱ. Surface plasmon coupling: constructing core-shell structures (such as Au@SiO_2_) or polymer enhanced local thermal field; Ⅲ. Multi functional integration: combining drug loading (such as doxorubicin), immune regulation (PD-L1 inhibitors), or photoacoustic imaging to achieve integrated diagnosis and treatment. The latest technologies currently include intelligent responsive Au NRs (such as pH/GSH triggered drug release) and improved tumor penetration and renal clearance through ultra small Au NPs (<5 nm). The future challenges also face long-term retention toxicity, uniformity in large-scale preparation, and limitations in tissue penetration depth. The key points for future development lie in accelerating clinical translation (such as combination immunotherapy), developing biodegradable Au based materials and optimizing photothermal performance with AI assisted morphology design.

### 2.4 Palladium nanosheets (PdNSs)

**FIGURE 5:**
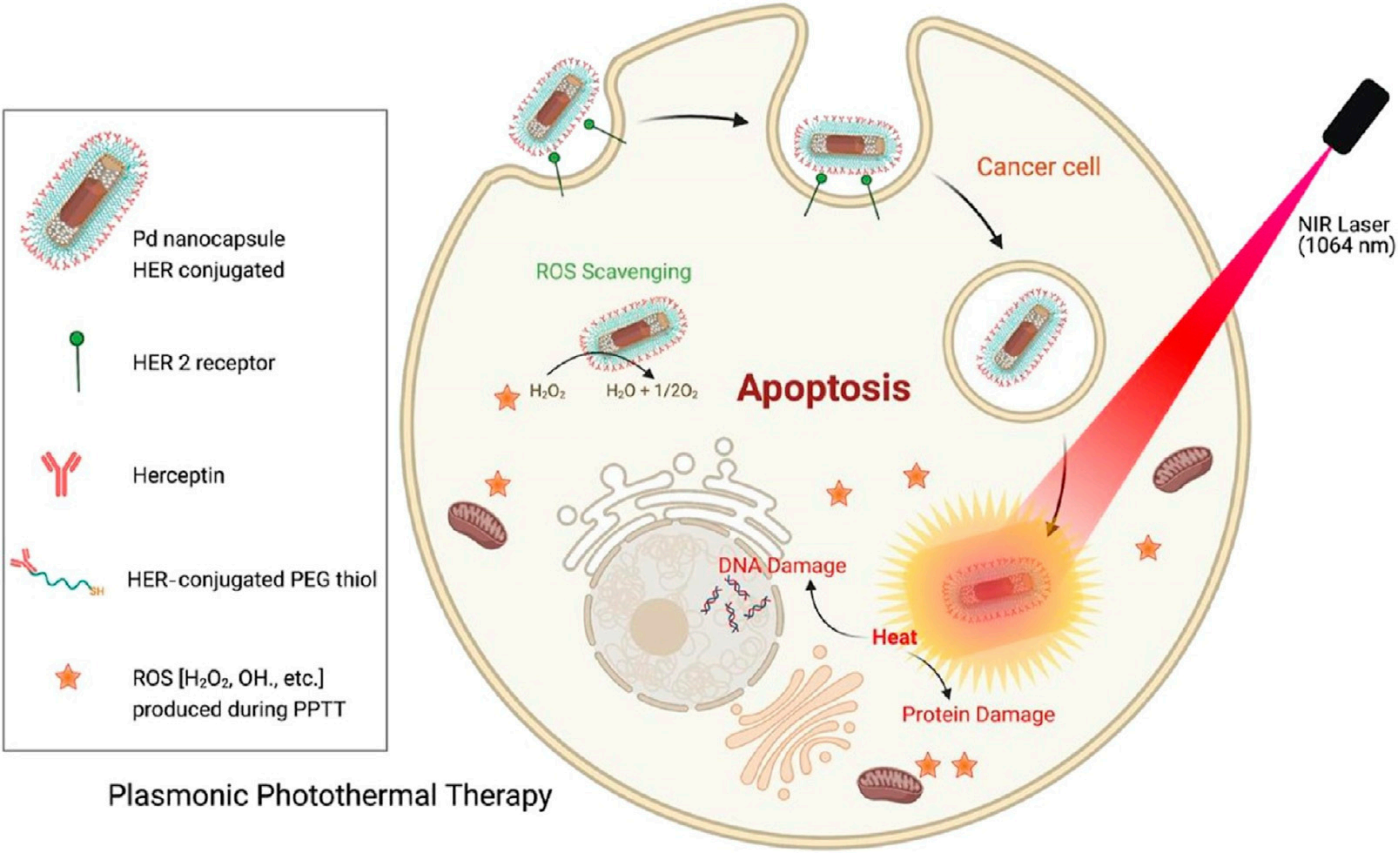
Schematic representation of the targeted plasmonic photothermal therapy of breast cancer cells using bimetallic herceptin-conjugated palladium nanocapsules (reprinted with permission from Singh et al., 2023. Copyright 2023 American Chemical Society).

PdNSs are thin and possess large specific surface area and planar size (Li et al., 2024). The planar surface allows for better absorption of light, as a unique advantage in PTT (He et al., 2022). The NIR-II response characteristics of Pd NSs are derived from their two-dimensional electronic confinement effect. The ultra-thin structure (thickness<2 nm) causes electrons to move freely in the plane, forming a wideband LSPR (1,000–1,350 nm), with a significantly higher photothermal efficiency (∼60%) than bulk Pd (<10%). The hot electrons generated by photoexcitation transition from d-band to sp band, and then release energy through electron phonon coupling. Unlike spherical NPs, the planar structure of Pd NSs endows them with unique membrane interaction modes (Li et al., 2022). The sharp edges of Pd NSs can physically damage the cell membrane and enter the cytoplasm directly through non endocytic pathways, avoiding lysosomal degradation. Pd NSs can inhibit key enzymes involved in tumor cell glycolysis, such as HK2 and LDHA, reduce ATP production, and enhance hyperthermia sensitivity (Chen et al., 2017). Figure 5 shows a schematic diagram of a bimetallic palladium nanocapsule (Pd Ncap) targetting the breast cancer cell line SK-BR-3 (Singh et al., 2023). Pd Ncap owns a rattle like morphology because of a solid gold bead as a core located inside a porous thin Pd shell. These nanostructures possess broad absorbance in the NIR biological windows (600–1,300 nm), thus enhancing their applicability in PPTT. PdNSs remain stable *in vivo* without aggregation or degradation, and they exhibit low biotoxicity (Yao et al., 2022). Additionally, PdNSs can be used in combination with other therapies, such as chemotherapy and immunotherapy (Li et al., 2022). For example, Jiang developed PdNSs Pd (5)-CpG (PS), which significantly enhanced the absorption of cytosine polyguanine (CpG) by immune cells and boosts the immune-stimulatory activity of CpG (Ming et al., 2020). Combined with Pd (5)-CpG (PS)-mediated PTT and immunotherapy, using safe NIR radiation (808 nm laser, 0.15 W cm^−2^), highly effective tumor inhibition and a significant increase in the survival rate of tumor-bearing mice were achieved. By surface modification, PdNSs can target tumor tissues (Yang et al., 2021). They cause local high temperature which disrupts the structure of diseased cells and interferes with their metabolism. Singh reported a bimetallic palladium nanocapsule (Pd Ncap) with a solid gold core and a thin, perforated palladium shell that demonstrated excellent photothermal stability (Singh et al., 2023). At a very low laser power density of 0.5 W cm^−2^, the PCE in 1,064 nm region reached 49%. The nanocapsules were further functionalized with Herceptin (Pd Ncap-Her) to target the breast cancer cell line SK-BR-3. *In vitro* PTT applications with NIR light, at a concentration of 50 μg/mL and a laser power density of 0.5 W cm^−2^ with an output power of only 100 mW, more than 98% of the cells were killed. Innovative designs and strategies for developing Pd NSs include: Ⅰ. Ultra thin structure optimization: regulating thickness (2–5 nm) and lateral size (50–200 nm) to enhance surface plasmon resonance (SPR) and improve photothermal conversion efficiency (>80%). Ⅱ. Multi functional composite: such as drug loaded composite catalytic function, utilizing the catalytic ability of Pd to decompose H_2_O_2_ and enhance CDT. Ⅲ. Heterogeneous structures (such as Pd@Au) improve photothermal stability and biocompatibility. Ⅳ. Surface modification: PEGylation or targeted molecule (such as RGD peptide) modification enhances tumor enrichment ability. The latest developed technologies currently include dual-mode therapy combining PTT/CDT or photothermal immunotherapy, and responsive drug release systems that utilize the tumor microenvironment (such as low pH/high GSH) to trigger drug release. The challenges of the material development still lie in the lack of long-term biosafety data and the control of size uniformity in large-scale synthesis. Future development can be achieved through the study of ultra-thin degradable Pd based nanosheets, combined with AI prediction of optimal morphology parameters to promote clinical translation.

### 2.5 Carbon nanomaterials

**FIGURE 6:**
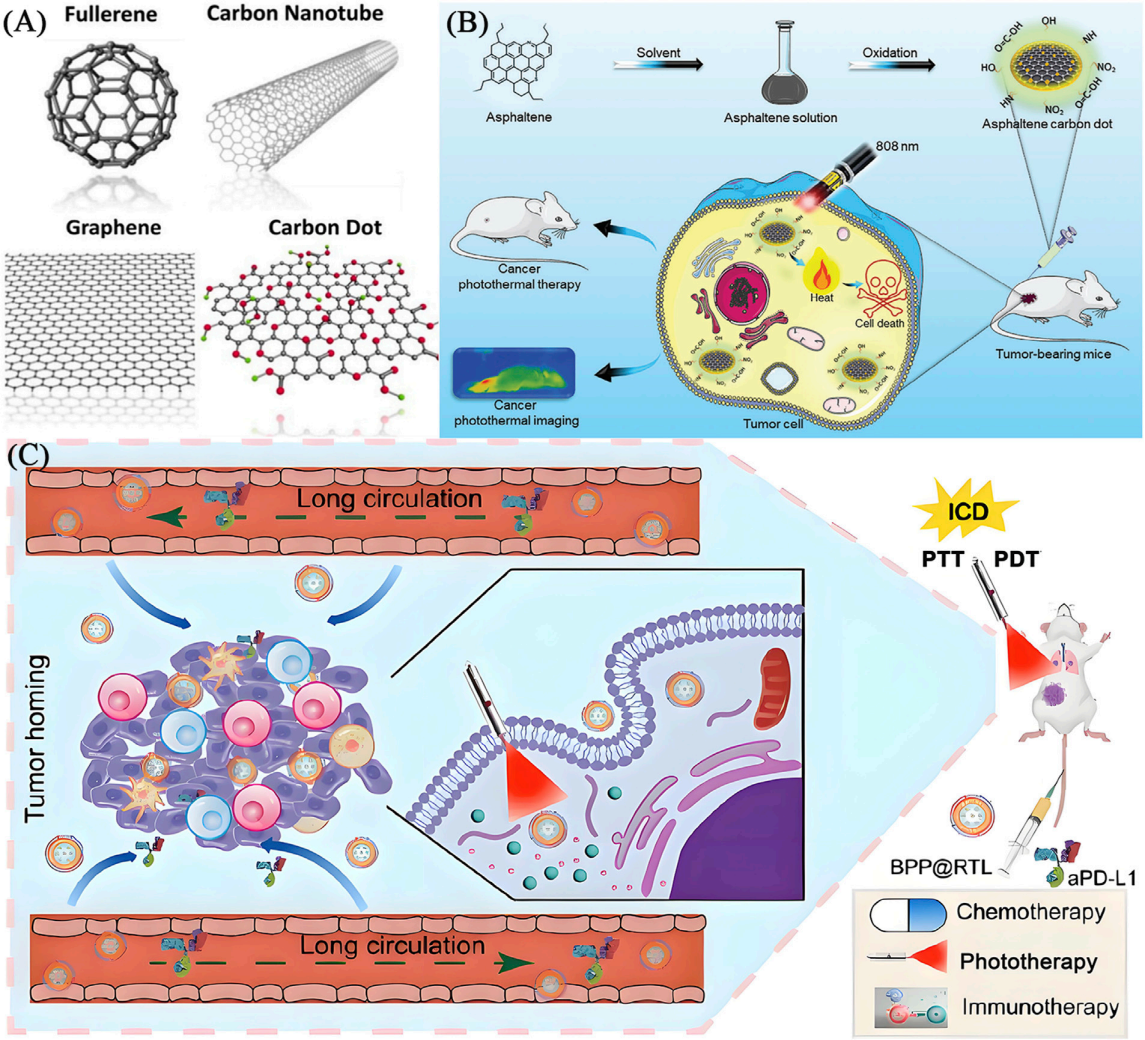
**(A)** The structures of carbon nanomaterials including carbon nanotube, graphene, fullerene and carbon dot (reprinted with permission from Hong et al., 2015. Copyright 2015 American Chemical Society). **(B)** Schematic illustration of ACDs synthesis from asphaltenes precursor and subsequent application in cancer photothermal therapy (reprinted with permission from Akakuru et al., 2025, licensed under CC BY). **(C)** Schematic image of fabrication and application of a QD-based nano-transformer (GQDNT) for diagnosis and treatment of cancer (reprinted with permission from Cao et al., 2024. Copyright 2024 American Chemical Society).

Carbon nanotubes (CNTs), carbon dots (CDs) and quantum dots (QDs) (Figure 6A) (Hong et al., 2015) achieve photothermal conversion through different molecular mechanisms. CNTs are plasmonic resonances excited by conjugated π electrons in NIR light, generating heat through electron phonon coupling (Liu et al., 2015). Their efficiency is regulated by diameter, chirality, and surface modification. CDs generate heat mainly through non radiative transitions between surface defect states and functional groups (such as amino groups), and doping can extend absorption to the NIR-II region (1000–1350 nm). QDs are controlled by quantum confinement effects to regulate their bandgap, electron hole pairs generate heat through Auger relaxation, and carbon based QDs combine with surface state effects to reduce toxicity (Sun et al., 2019). CNTs rely on clathrin endocytosis to disrupt lysosomes. CDs/QDs regulate endocytosis efficiency through size and charge regulation, while utilizing targeted modifications (such as folate) to target mitochondria, enhancing tumor specificity. CNTs/CDs/QDs can interfere with DNA repair in tumor cells and induce protein denaturation, ROS burst, and apoptosis/necrosis through local temperature rise (42°C–48°C). The PTT performance of carbon materials is highly dependent on their defect density - moderate oxidation treatment (C/O ratio≈8:1) can optimize absorption spectra while maintaining stability. Figures 6B,C shows the typical schematic diagrams of using CDs and QDs for PTT (Li J. et al., 2021; Cao et al., 2024).

#### 2.5.1 Carbon nanotubes (CNTs)

**FIGURE 7:**
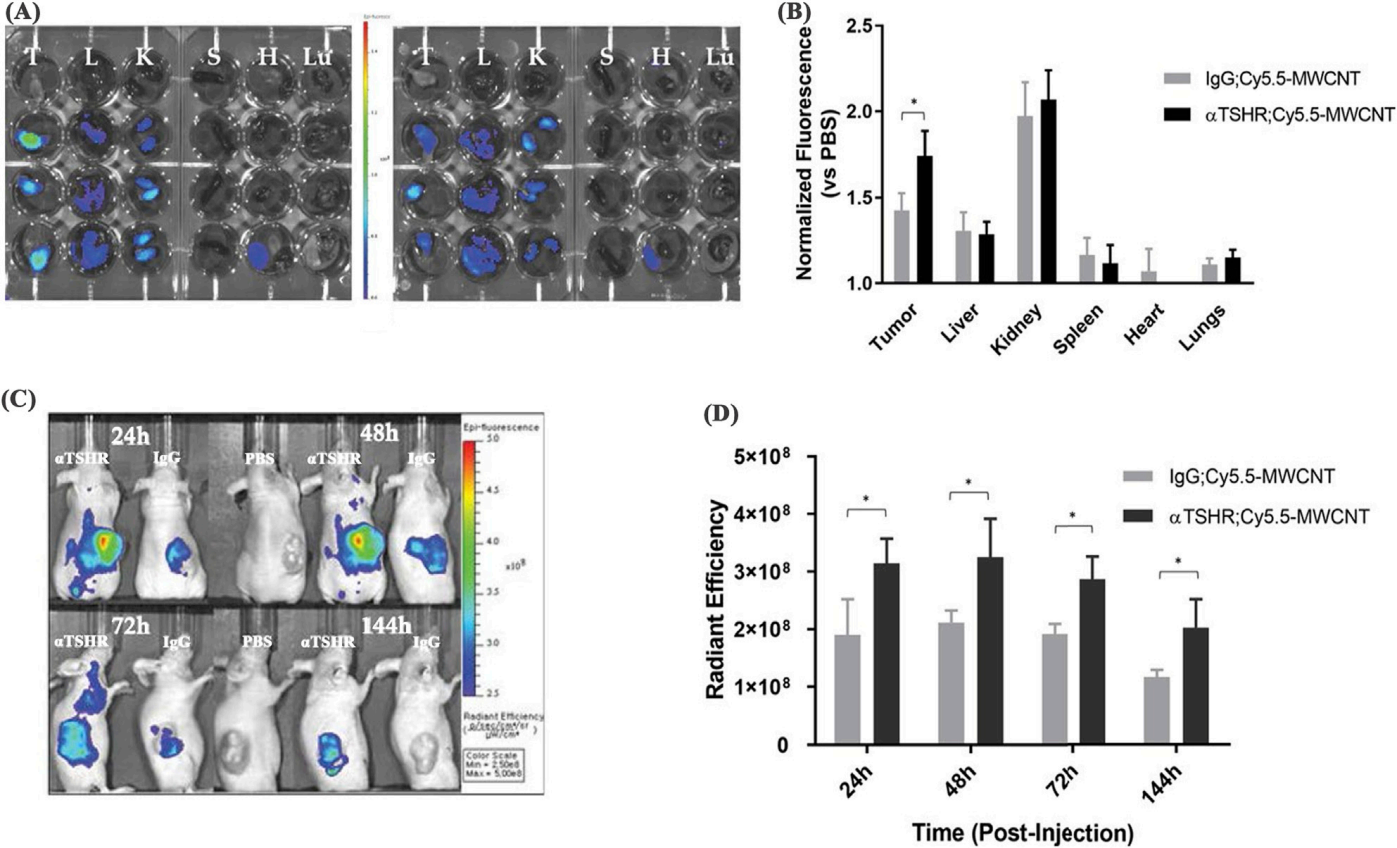
*In vivo* biodistribution of αTSHR-Cy5.5-MWCNT. **(A)** IVIS images of major organs (T = tumor, L = liver, K = kidney, S = spleen, H = heart, Lu = lung) 24 h post-injection. **(B)** Tumor accumulation showed 1.22-fold increase for αTSHR-Cy5.5-MWCNT vs. IgG-Cy5.5-MWCNT (p = 0.0376), with no significant changes in other organs. **(C)** Weekly tumor accumulation profile. **(D)** Time-course showing enhanced tumor targeting by αTSHR-Cy5.5-MWCNT (p = 0.046, 0.049, 0.019, and 0.043 at 24, 48, 72, and 144 h, respectively) (reprinted with permission from Lee et al., 2023, licensed under CC BY 4.0).

Depending on the number of graphene layers, CNTs are classified into single-walled carbon nanotubes (SWCNTs) and multi-walled carbon nanotubes (MWCNTs) (Sajja et al., 2021). SWCNTs consist of sp-hybridized carbon atoms arranged in a hexagonal honeycomb lattice, forming a hollow tubular structure (Afroze et al., 2021), while MWCNTs are composed of multiple concentric tubes nested within each other. CNTs exhibit strong absorption in the near-infrared (NIR) spectrum and PCE (Farbod et al., 2024). In addition, functionalization of the CNT surface with targeting ligands can reduce the toxicity and immunogenicity (Mohanta et al., 2019), which makes CNTs highly suitable for PTT and PDT. The high aspect ratio and specific surface area enable CNTs to adsorb a variety of drug molecules (Zhao et al., 2022). Their needle-like structure facilitates internalization into target cells, which could be an advantage for drug delivery (Naief et al., 2024). The PTT with CNTs has primarily focused on relatively small primary tumors, but the approach has limitations in preventing tumor metastasis. To overcome this, Mc Kernan developed SWCNT-ANXA5 by modifying SWCNTs with annexin A5 (ANXA5) (McKernan et al., 2021). Under NIR irradiation, this bioconjugate selectively ablated primary orthotopic EMT6 breast tumors in mice, synergistically enhancing the T lymphocyte-associated protein-dependent abscopal response, thus inhibiting tumor metastasis. As a result, the survival rate of mice treated with PTT was significantly extended, with survival persisting up to 100 days after tumor inoculation. Guo prepared an optically and thermally stable multi-walled CNT-hyaluronic acid composite (MWCNT-HA) (Guo et al., 2023). MWCNT-HA upregulates the expression of the apoptotic factor Caspase-3, which in turn affects the expression of the downstream anti-apoptotic factor Bcl-2, leading to apoptosis in CNE-1 cells, thereby inhibiting tumor cell proliferation and promoting apoptosis. Additionally, this material significantly raises the surrounding temperature under laser irradiation. When applied MWCNT-HA to tumor cells in mice, assisted by NIR laser irradiation, cell viability decreased by over 80% within 3 days. MWCNT-HA also greatly enhances the biosafety and compatibility of the drug, improving the therapeutic efficacy. Lee synthesized MWCNTs conjugated with thyroid-stimulating hormone receptor antibodies (TSHR), which selectively accumulate in tumor sites and remain for extended time (Figures 7C,D) (Lee et al., 2023). In BCPAP xenograft mice, αTSHR-Cy5.5-MWCNT (1 mg/kg, i.v.) showed predominant tumor accumulation at 24 h, with significantly enhanced targeting (p = 0.038 vs. IgG-Cy5.5-MWCNT) (Figures 7A,B). This tumor-specific distribution guided subsequent laser treatment timing. Upon laser irradiation, the conjugates induced tumor cell ablation, minimizing non-specific damage, which demonstrates a strong cytotoxic effect on thyroid tumor cells, inhibiting their regeneration and delaying tumor recurrence.

#### 2.5.2 Carbon dots (CDs)

CDs possess a large π-conjugated system with a core composed primarily of graphitized sp^2^ carbon (Huo et al., 2021). Their shell contains abundant functional groups, such as carboxyl, hydroxyl and amine groups, which bring excellent water solubility and facilitate the combination of CDs with therapeutic agents (Lagos et al., 2023). Wang synthesized copper-doped Cu-CDs using urea and ethylene glycol as carbon sources and copper sulfate as an active dopant, employing one-step hydrothermal method (Wang et al., 2024). Cu-CDs effectively inhibited the proliferation of breast cancer cells (MDA-MB-231) by disrupting their malignant behavior. CDs can convert NIR photons into heat and promote the production of reactive oxygen species (ROS) in the surrounding environment, leading to cell apoptosis and achieving tumor treatment. Kim synthesized sulfur-doped CDs (S-CDs) with strong NIR absorption, achieving a photothermal conversion efficiency of 55.4% (Kim et al., 2021). When injected at a low dose (45 μg/mL) and combined with moderate laser irradiation (808 nm, 1.1 W cm^−2^), S-CDs could completely ablate cancer cells with minimal side effects. CDs possess tunable size and structural units, small volume, modifiability, good biocompatibility and low toxicity. For example, Bao designed PEG-modified Cu-CD cross-linked nanosheets, which effectively killed MCF-7 cells without exhibiting toxicity towards healthy cells (Bao et al., 2018). Additionally, Phuong developed a highly selective and sensitive PTT using CDs for mitochondrial-targeted cellular imaging (Phuong et al., 2020). The CDs were combined with TiO_2_ (C-CD/TiO_2_) to create a pH-responsive system that precisely targeted the cell membranes and nuclei of acidic cancer cells, effectively ablating the cancer cells and upregulating pro-apoptotic markers within them. This system demonstrated excellent targeting abilities for both bio-imaging and therapy. These advantages enable functional CDs to serve as drug carriers.

#### 2.5.3 Quantum dots (QDs)

QDs are constrained in three spatial directions. QDs exhibit excellent optical stability and biocompatibility (Tade and More, 2025). The surface of QDs contains oxygen-rich functional groups such as hydroxyl and carboxyl, which are soluble in water and facilitate the application of QDs in biological systems. QDs also possess NIR absorption properties, allowing them to generate photothermal effect (Zhan et al., 2022). During localized hyperthermia, QDs promote the generation of ROS, so QDs are suitable for synergistic PTT and PDT. Tian synthesized DOX-ZIF-8/GQD, which showed significant temperature increases under NIR radiation (Iannazzo et al., 2017). The nanoparticles deeply penetrate biological tissues and be absorbed by tumor tissues, where they generate localized hyperthermia to kill tumor cells. QDs have a high surface area-to-volume ratio, providing more active interactions with the surrounding environment, which is also crucial for their adsorption ability (Yang et al., 2023). By linking specific ligands or antibodies to the surface of QDs, they can bind specific receptors to tumor cells, achieving active targeting. For instance, Zhong developed highly water-soluble (104 μg mL^−1^ at 25°C), highly luminescent (quantum yields of 77%) graphene QD with folate receptor targeting using folic acid as a precursor (Khodadadei et al., 2017). These QDs demonstrated excellent fluorescence labeling and targeted binding to breast and ovarian cancer cells expressing different levels of folate receptors, without exhibiting cytotoxicity. Interestingly, although the targeting mechanism is mediated by folate receptor endocytosis, the QDs do not bind through the pterin-folate receptor interaction. Instead, after thermal decomposition of the pterin ring on the folate molecules, residues remain on the surface of the QDs, interacting with organic functional groups and promoting specific binding to folate receptors on tumor cells, achieving targeted selectivity. Additionally, the structure of QDs allows them to own higher drug loads, making them more efficient in delivering chemotherapeutic agents. The π-orbitals in the sp^2^-hybridized QD lattice can also stack with aromatic rings in chemotherapeutic drugs and enhance drug delivery without covalent conjugation (Zhao et al., 2024b).

The above indicates that the functional efficiency of carbon nanomaterials is usually improved through structural optimization, doping with heteroatoms and multifunctional composites. Such as reducing graphene oxide (GO) to regulate the sp^2^/s^3^ carbon ratio and enhance the photothermal conversion efficiency (>40%), hollow carbon spheres or mesoporous carbon enhance drug loading capacity, nitrogen/sulfur doped carbon dots (N-CDs) enhance NIR absorption and catalytic activity, achieving PTT/CDT synergistic therapy, carbon based carrier loaded metal nanoparticles (such as Au@C) enhance photothermal effect, surface modify targeted molecules (such as folate) to improve tumor selectivity and so on. The latest emerging technologies include carbon nano intelligent response systems, such as pH/photothermal dual controlled drug release and ultra small carbon based quantum dots (<10 nm) to improve tumor penetration. The development bottleneck is the unclear long-term metabolic mechanism in the body and the batch stability of large-scale preparation of carbon nanomaterials. Developing biodegradable carbon based materials in the future and combining them with AI to optimize the band structure will promote clinical translation.

## 3 Low-temperature-driven multidimensional antitumor paradigm

The key to low-temperature PTT lies in how it is carried out at relatively low temperatures (usually less than 42°C). Its advantage lies in the ability to inhibit the synthesis of heat shock proteins, reduce the heat resistance of tumor cells, thereby improving the effectiveness of PTT and reducing the temperature required for treatment. This has important value for the future clinical translation of cancer PTT (Yan et al., 2024). Mild thermal stress (42°C–45°C) can selectively lead ICD in tumor cells, release DAMPs (such as CRT, HMGB1, ATP), promote DCs maturation and initiate CTLs responses. Taking PDA as an example, its low-temperature photothermal effect (ΔT ≈ 8°C–10°C) not only avoids upregulation of HSP70, but also activates the cGAS STING pathway through LMP, driving type I interferon secretion and transforming “immune cold tumors” into T cell enriched phenotypes. This “heat immune synergy” effect breaks through the local limitations of traditional PTT and can significantly prolong survival when combined with immune checkpoint inhibitors such as anti-PD-1. Abnormal metabolism of tumor cells, such as the Warburg effect, leads to microenvironmental acidification and immune suppression, and low-temperature PTT can break this vicious cycle through metabolic intervention. For example, under NIR-II irradiation, Pd NSs block the glycolytic pathway by inhibiting hexokinase 2 (HK2), reduce lactate production, reverse M2 polarization of TAMs, and reduce the expression of heat tolerant proteins, thereby increasing the sensitivity of tumor cells to sublethal temperature rise by 3–5 times. This dual targeted strategy of “metabolism hyperthermia” provides a new approach to overcome tumor heterogeneity (Uson et al., 2022).

To achieve precise low-temperature PTT, a new generation of intelligent materials (such as pH/ROS dual responsive semiconductor nanoparticles) are activated specifically by the lesion microenvironment, strictly limiting thermal effects to the tumor area (spatial accuracy<200 μm) to avoid off target damage. For example, CuS@MnO The core-shell structure generates heat *in situ* under acidic and high H_2_O_2_ conditions, resulting in a temperature gradient of 6°C–8°C between tumor and normal tissue (Jin et al., 2024). However, clinical application still faces the following challenges: lack of material biodegradability and long-term toxicity assessment system; The dose-response relationship between the low-temperature effect and immune response is unclear; The establishment of scientific standards for large-scale preparation processes and supervision is urgently needed. This review calls for the establishment of three-level evaluation indicators to replace traditional tumor inhibition rates: (1) immunogenicity index (such as CD8^+^T cell infiltration rate); (2) Depth of metabolic regulation (such as changes in lactate/ATP ratio); (3) Induction rate of remote effect. In addition, exploring the cross fusion of low-temperature PTT with epigenetic regulation (such as HDAC inhibitors) or microbial therapy may open up a new dimension of “photothermal microbiome immune” multidimensional therapy. This paradigm shift not only drives PTT from “rough thermal ablation” to “precise immune metabolism regulation,” but also offers a multi-scale mode for personalized tumor treatment.

## 4 Loading strategy and application of photothermal agents on nanocarriers

Various integrated nanoplatforms have been developed by loading photothermal agents onto multifunctional nanocarriers or combining them with other therapeutic molecules, effectively overcoming the limitations of single PTT (such as insufficient penetration depth, drug resistance, off target damage). This section focuses on the strategies and application progress of PDA, SNPs, AuNPs, palladium nanosheets and carbon materials as nanocarriers for loading photothermal agents or constructing composite systems. Based on the physical and chemical properties such as surface functional groups, porosity and plasma resonance effects, different nanocarriers can adopt the following strategies to achieve efficient loading and functional synergy of photothermal agents: Ⅰ. Physical adsorption: Utilizing π - π stacking, hydrophobic interactions, or electrostatic adsorption to load small molecule photothermal agents (such as indocyanine green ICG) or drugs. For example, PDA nanoparticles load doxorubicin (DOX) through π-π interactions, achieving photothermal triggered drug release (pH/NIR dual response) (Wang et al., 2022). Ⅱ. Chemical coupling: Fixing targeted ligands or photosensitizers on the surface of a carrier through covalent bonds (such as thiol gold bonds, amino carboxyl condensation). For instance, surface modification of AuNPs with HA targets tumor cells overexpressing CD44, and covalently Ce6 to achieve photothermal/photodynamic synergistic therapy (Liu et al., 2022). Ⅲ. Encapsulation: Co-loading of photothermal agents and drugs is achieved through mesoporous structure, core-shell design or liposome encapsulation. Such as mesoporous CuS@SiO NPs coated with DOX utilize NIR-II to trigger drug release and enhance deep tumor killing (Li et al., 2022). Ⅳ. Composite hybridization: Combining with other nanomaterials such as metal organic frameworks (MOFs) and graphene to enhance photothermal conversion efficiency or introduce catalytic functions. Table 3 summarizes the loading strategies, synergistic therapeutic mechanisms and application cases of five types of nanomaterials. Although loaded nanocarriers have shown great potential, some key issues still need to be addressed, such as loading efficiency and stability, penetration depth limitations, metabolism and toxicity, and clinical translation bottlenecks. In the future, intelligent responsive carriers will be developed to achieve precise drug release. Build an integrated PTT immunotherapy platform by combining mRNA vaccines or CAR-T cell therapy. Using AI assisted design of nanocarrier structures to optimize photothermal conversion efficiency and biological distribution.

**TABLE 3:** The loading strategies, synergistic therapeutic mechanisms and application cases of five types of nanomaterials.

Nanocarriers	Loading strategies	Composite systems	Synergy mechanisms	Application cases
Polydopamine (PDA)	Physical adsorption (drug/ICG) Chemical coupling (targeting peptide)	PDA@DOX-ICG (Ferreira et al., 2021) PDA-RGD-Ce6 (Li et al., 2023c)	pH/NIR dual responsive drug release; Photothermal enhancement of tumor permeability and immunogenicity	Triple negative breast cancer (DOX/PTT under NIR-I excitation, inhibit lung metastasis)
Semiconductor NPs (SNPs)	Chemical coupling (targeting peptide) Surface modification (antibody)	P@GMT-R (Li et al., 2022e) SPN-PT (Yuan et al., 2022)	NIR-II photothermal triggered drug release; Reversal of immunosuppressive microenvironment	Orthotopic osteosarcoma(NIR-II penetrating the blood-brain barrier, combined with immunotherapy)
Au Nanomaterials	Gold sulfur bond coupling (photosensitizer) Core-shell coating (drug)	AuNRs-Ce6 (Chuang et al., 2020) AuNR@PAMAM-GX1 (Ye et al., 2021)	Plasma resonance enhances ROS generation; Photothermal/chemotherapy/photodynamic three mode treatment	Head and neck squamous cell carcinoma (gold nanorods combined with Ce6 to achieve in situ ablation and distal effect)
Palladium nanosheets	Surface adsorption (small molecule drug) Composite catalytic material	Pd@Gemcitabine (Wang et al., 2020a) CuGQD/PdNPs@PSi (Zhao et al., 2023b)	Photothermal enhanced drug internalization; Catalytic decomposition of H2O2 to produce oxygen to alleviate tumor	Hypoxia pancreatic cancer (gemcitabine loading combined with catalytic therapy)
Carbon Nanomaterials	Covalently modified (targeted molecules) Composite magnetic particles	Graphene-QDs/DOX (Wang et al., 2020b) DOX @GQDs-PAMAM-β-CD (Pooresmaeil et al., 2020)	Photothermal/magnetocaloric synergistic heating; Carbon based carrier enhances drug loading stability	Melanoma (graphene quantum dots combined with magnetocaloric therapy to achieve bimodal imaging guided PTT)

## 5 Conclusion and outlook

In recent years, functional nanomaterials have made progress in the field of tumor PTT, providing strategies to break through the limitations of traditional cancer treatment. By combining efficient photothermal materials with functional modification, researchers have successfully developed nanodelivery system with targeted delivery, multimodal imaging, synergistic therapy and so on. The materials can achieve local high-temperature killing tumor cells under NIR light excitation, while significantly improving tumor enrichment efficiency and reducing damage to normal tissues through surface functionalization (such as antibody and peptide modification). In addition, the synergistic application of nanomaterials with chemotherapy, radiotherapy and immunotherapy further amplifies the anti-tumor effect, such as activating the systemic anti-tumor immune response through photothermal induced immunogenic cell death (ICD), providing the possibility to inhibit metastasis and recurrence. Preclinical studies have confirmed that some nanosystems exhibit significant advantages in penetration depth, photothermal stability and biosafety, laying the foundation for translational medicine. However, the clinical translation of functional nanomaterials still faces multiple challenges. Firstly, the long-term biocompatibility and metabolic pathways of nanomaterials are not yet clear, and some metal based materials have potential toxicity risks, which urgently require the development of degradable or biologically inert carriers. Secondly, tumor heterogeneity and complex microenvironments (such as hypoxia and acidic pH) may weaken PTT therapy, requiring the design of intelligent responsive nanomaterials to achieve on-demand release of energy or drugs. Again, existing research has mostly focused on small animal models, and the depth of light penetration, thermal diffusion effect and large-scale preparation process of human tissues still need to be systematically optimized. In addition, the targeting efficiency of nanomaterials is limited by the accuracy of tumor vascular permeability and surface modification, and real-time treatment monitoring needs to be achieved by combining molecular imaging technology. Future research directions include the followings: Ⅰ. Material innovation, developing ultra efficient photothermal agents with NIR-II response and utilizing theoretical calculation to accelerate material design; Ⅱ. System integration, building an integrated diagnosis and treatment platform, integrating photoacoustic imaging, photothermal/photodynamic/immunotherapy synergistic therapy functions; Ⅲ. Clinical adaptation, evaluating individualized treatment plans through organoid or patient derived xenograft (PDX) models; Ⅳ. Deepen the mechanism and analyze the molecular correlation between tumor cell death and immune microenvironment remodeling under photothermal stress. With the integration of interdisciplinary technologies and the improvement of translational medicine systems, functional nanomaterials are expected to promote tumor PTT from the laboratory to clinical practice, opening up new paths for precision oncology.
